# Workplace Bullying in a Sample of Italian and Spanish Employees and Its Relationship with Job Satisfaction, and Psychological Well-Being

**DOI:** 10.3389/fpsyg.2015.01912

**Published:** 2015-12-15

**Authors:** Alicia Arenas, Gabriele Giorgi, Francesco Montani, Serena Mancuso, Javier Fiz Perez, Nicola Mucci, Giulio Arcangeli

**Affiliations:** ^1^Department of Social Psychology, University of SevilleSeville, Spain; ^2^Department of Human Sciences, European University of RomeRome, Italy; ^3^Montpellier Business SchoolMontpellier, France; ^4^Institute of Public Health, Catholic University of the Sacred HeartRome, Italy; ^5^Health Services Research Unit, Department of Experimental and Clinical Medicine, University of FlorenceFirenze, Italy

**Keywords:** workplace bullying, cultural comparison, prevalence, psychological well-being, job satisfaction

## Abstract

Purpose – The purpose of this study is to examine the prevalence rate of workplace bullying in a sample of Italian and Spanish employees, and its differential consequences on employees’ job satisfaction and psychological well-being. The effects of workplace bullying on job satisfaction and psychological well-being were explored taking into account a contextualized approach. Design/Methodology/approach – Cross-sectional study was adopted, in which a sample of 1,151 employees in Italy and 705 in Spain completed a questionnaire. We hypothesized that the relationship between exposure to bullying behaviors and psychological well-being is mediated by job satisfaction, and that this simple mediation model is moderated by the country (moderated mediation). Findings – Results suggest that no particular differences exist in bullying prevalence among Spanish and Italian employees. However, we found scientific confirmation of our hypothesized moderated mediation model. Research limitations/implications – Despite the limitations of the sample studied, findings capture contextual differences in the bullying phenomenon, which may have several implications for further research in this domain, as well as for designing interventions to deal with workplace bullying. Originality/value – Although this study explores bullying in different cultural contexts without investigating specific cultural values, it establishes the roots to assess workplace bullying from a contextualized perspective.

## Introduction

Workplace bullying refers to an interpersonal process by which one individual ends in a helpless situation after being the target of systematic and subtle negative acts. It has severe consequences for employees and the organization as a whole and has situated workplace bullying as a prominent subject in research agenda during recent years (Matthiesen and Einarsen, 2010). Furthermore, several authors have considered workplace bullying as one of the main problems for workers’ safety and health because of its important prevalence and its severe consequences (e.g., Einarsen et al., 2011).

However, few comparison studies have been conducted in this domain, although some scholars have argued that the antecedents and consequences of workplace bullying cannot be analyzed without taking into account the differences in industrial relations and cultural values among countries and organizations (Chappell and Di Martino, 2006; Giorgi, 2010; Escartín et al., 2011; Giorgi et al., 2013).

Consequently, the present paper aims to examine workplace bullying by comparing its prevalence and consequences on employees’ job satisfaction and psychological well-being among two samples of Spanish and Italian employees. In particular, we expect to find a similar prevalence of bullying in the two samples because the literature shows that, in southern Europe, tolerance and acceptance of negative acts are higher than northern European countries (Parent-Thirion et al., 2007). Indeed, national culture may play a crucial role in the perception of bullying by employees (Escartín et al., 2011; Power et al., 2013) and bullying behaviors are, in Spain and in Italy, mainly top-down processes, in which the target is usually in a lower position than the perpetrator (Moreno Jiménez et al., 2008; Giorgi et al., 2011). In addition, in some jobs (e.g., among nurses), bullying may even be considered as part of the job, with tolerance and failure to report the episodes (Arcangeli et al., 2014).

### Prevalence of Workplace Bullying

According to the Fifth European Working Conditions Survey (EWCS: EUROFOUND, 2010), the prevalence of workplace bullying was estimated in 1.6% of the working population in the EU. However, this prevalence varied dramatically between countries, oscillating between 9.5% in France and 0.6% in Bulgaria. Since the method to estimate the prevalence of workplace bullying was the same across the countries that participated in the survey – that is, asking employees directly whether or not they considered they had been subjected to bullying over the past 12 months- it seems reasonable to think that personal and cultural factors might explain these vast differences.

Indeed, this estimation method may be biased, since the bullying prevalence can be underestimated in countries where the phenomenon is not yet well-known to the audience and its knowledge is partial or faulty, such as in Italy or in Spain (Moreno Jiménez et al., 2008; Giorgi et al., 2011). Thus, many targets of bullying can be unaware of the fact of being bullied as they really do not know what bullying is and what kind of behaviors it involves.

Therefore, it is not surprising that results from the Fifth European Working Conditions Survey have shown a higher prevalence of workplace bullying in Northern and Western European countries compared to Southern and Eastern European countries, since the former are pioneers in the study of this phenomenon and there is a higher awareness of bullying and its severe consequences, whereas bullying has only been of recent interest or is just starting to be considered a social problem in the majority of Southern and Eastern European countries (Giorgi et al., 2011). Several theoretical and methodological shortcomings have been argued to explain these notable differences in bullying prevalence (Parent-Thirion et al., 2007). Furthermore, results from the European Survey of Enterprises on New and Emerging Risks (ESENER) conducted in 2009 (Rial-Gonzalez et al., 2010) seem to support this “awareness hypothesis” regarding bullying prevalence. Many authors have suggested that this awareness could make employees more prone to perceive and recognize the issue, compared to Southern European countries, like Italy, in which knowledge of the relevance and the dangers of bullying is limited (Salin, 2001; Escartín et al., 2011; Power et al., 2013).

The ESENER survey asked managers and workers’ health and safety representatives of 31 European countries about how health and safety risks are managed at their workplace. Results showed that, in Spain, only 17.64% of the surveyed employers indicated that there was a formal procedure in their organizations to deal with workplace bullying, and this percentage dropped to 9.43% in Italy. On the other hand, at least a half of the organizations in Northern European countries have formal procedures to tackle bullying, according to participants’ responses (80.41% UK, 71.59% Sweden, 62.78% Finland, and 55.43% Norway). In conclusion, it is possible that the higher rates of bullying prevalence in Northern European countries are simply highlighting more awareness and sensitivity about the problem in the society than in Spain or Italy.

In addition, taking together that bullying is mainly a top-down process in Spain and Italy, in which the target is usually in an inferior hierarchical position than the perpetrator (Moreno Jiménez et al., 2008; Giorgi et al., 2011), and the fact that, in some cultures, especially in masculine cultures (c.f., Hofstede, 2001), bullying may be considered as part of the job, or as a reasonable managerial practice (e.g., Hoel et al., 2004; Escartín et al., 2009, 2011), it seems consistent that employees are more likely to tolerate negative acts in Spain and Italy than in other cultures where people are better able to recognizing bullying episodes and are more ready in reporting them (Parent-Thirion et al., 2007; Moreno Jiménez et al., 2008).

Consequently, as these findings suggest that cultural and individual sensitivity toward bullying may introduce bias when the phenomenon is addressed, several researchers consider that using a bullying behavior inventory without explicitly referring to the bullying construct, and following an operative criteria of being exposed to a certain number of bullying behaviors or negative acts on a weekly or daily basis during the last 6 months, avoids priming effects and facilitates international comparisons (Einarsen et al., 2009; Nielsen et al., 2010, 2011).

Furthermore, we hypothesized that there will not be significant differences in the prevalence of workplace bullying among two samples of Spanish and Italians workers if an operative criteria method is used, since: (a) using an operative criterion method avoids the possible influence of being more or less aware of what bullying is (Einarsen et al., 2009; Nielsen et al., 2011); (b) previous studies conducted in Italy and Spain found a higher prevalence of bullying when comparing with Northern European countries (Giorgi et al., 2011); (c) a similar number of both Italian and the Spanish health and safety workers’ representatives that participated in the ESENER survey indicated that they had received requests to tackle bullying during the period 2006–2009 (17.7% in the case of the Spanish representatives and 15.1% in the case of the Italian representatives); and (d) the exposition of bullying behaviors may be not significantly different if cultural variables are considered, since, according to Hofstede’s framework, Italy and Spain score similarly in power distance and uncertainty avoidance as well as, according to Schwartz (2006, 2007), also having similar cultural value orientations.

H1: There will be similarities in the prevalence of workplace bullying among two samples of Spanish and Italian employees if an operative criteria method is used.

### Consequences of Workplace Bullying

A considerable amount of research evidence suggests that workplace bullying has severe negative consequences for employees’ health and well-being, for organizational performance, and even for the social context (Mikkelsen and Einarsen, 2001; Hoel et al., 2004; Bowling and Beehr, 2006; Topa-Cantisano et al., 2007; Hogh et al., 2010). Focusing on an individual level, it is agreed that bullying is associated with decreased job satisfaction, deterioration of psychological well-being and increased job-related stress, which may cause physical symptoms and psychological disorders in the victim (Djurkovic et al., 2006; Rodríguez-Muñoz et al., 2009; Einarsen et al., 2011; Lovell and Lee, 2011). Bullying is often associated with psychological distress and psychosomatic symptoms, including burnout (Einarsen et al., 1998; Savicki et al., 2003). In some studies, it has been investigated as to how exposure to workplace bullying may weaken psychological health and findings suggest that workplace bullying negatively predicts job performance and positively predicts burnout (Sà and Fleming, 2008; Trépanier et al., 2013). Trépanier et al. (2013), on the basis of self-determination theory, experienced a model in which workplace bullying predicted a condition of poor psychological health and well-being related to a lack of satisfaction of basic psychological needs (autonomy, competence, and relationships). The results showed that workplace bullying negatively predicted job performance – in relation to the lack of autonomy, competence and relationships – and positively predicted burnout – in relation to the lack of autonomy (Trépanier et al., 2013).

Thus, we hypothesize that higher exposure to bullying behaviors will be related to less perceived job satisfaction and psychological well-being.

H2: A higher exposure to bullying behaviors will be associated with less psychological well-being in both samples.
H3: A higher exposure to bullying behaviors will be associated with less job satisfaction in both samples.

In addition, some authors consider workplace bullying as a serious stressor at work (Bowling and Beehr, 2006; Baillien et al., 2011). In that sense, various factors play a buffering role in the relationship between workplace bullying and psychological well-being (relationship stressor-strain), as, for example having certain personal characteristics and personality (e.g., Djurkovic et al., 2006; Glaso et al., 2007), or having social support and a good organizational climate (e.g., Fairbrother and Warn, 2003; Muñoz et al., 2006; Giorgi, 2010).

As “job satisfaction refers to a pleasurable or positive emotional state resulting from the appraisal of one’s job experiences” (Rodríguez-Muñoz et al., 2009, p. 228), which is quite stable over time (Dormann and Zapf, 2001), and taking into account that, after conducting a meta-analysis, Faragher et al. (2005) concluded that “job satisfaction level is an important factor influencing the health of workers” (Faragher et al., 2005, p. 105), it seems reasonable to think that satisfaction with different job dimensions, such as the salary/wage or the relationship with workmates, may be also conceived as an emotional resource to deal with bullying that mediates the consequences of bullying at work on employees’ health and well-being.

Nevertheless, job satisfaction differs among countries (e.g., Sousa-Poza and Sousa-Poza, 2000). For instance, in the European Quality of Life Survey (EQLS, EUROFOUND, 2007, 2012) Spanish employees were found to be more satisfied than Italian employees. In addition, life satisfaction is moderately correlated with mental health and working conditions (EUROFOUND, 2012). Thus, one would expect that the relationship between workplace bullying and psychological well-being may be mediated by job satisfaction, but also the relationship between job satisfaction and psychological well-being could be different depending on the country (moderation effect). We expect this effect in consideration of the differences in the cultural values across the two samples, as suggested by Hofstede (2001): it seems that Italians score higher in masculinity and individualism, whereas Spanish score higher in Indulgence. This difference could suggest indirect effects depending on the country of origin. People in most countries rate their level of well-being higher than their level of satisfaction with life in general.

H4: Country differences in job satisfaction will influence the mediating effects of job satisfaction on the relationship between workplace bullying and psychological well-being (moderated mediation model).

To sum up, this study examines the prevalence rate of workplace bullying in two samples of Spanish and Italian employees. Data from previous studies conducted in Italy and Spain (Moreno Jiménez et al., 2008; Giorgi et al., 2011) revealed low rates in comparison with Northern European countries when subjective estimation methods are considered. In contrast, when objective measures (such as the NAQ-R) were used, bullying prevalence was very high. In this context, our research may lead to an understanding of the characteristics of the issue in a sample of Italian and Spanish employees, whereas most of the studies were conducted in the countries of northern Europe. In addition, we used an operational approach in which participants had the opportunity of indicating how frequently they were exposed to potential bullying behaviors or negative acts. Such an approach may provide more “objective” estimates of the prevalence of bullying as based on the Bergen criterion (operational method). It is worth noting that the use of valid and reliable tools to assess workplace bullying is of utmost importance, especially in countries (such as Spain and Italy) where there is a limited awareness of the issue.

## Materials and Methods

### Procedure and Participants

Data were collected by means of paper and pencil questionnaires. Informed consent was obtained from each subject of the research. Ethics committees of both an Italian and a Spanish university approved the study.

In Italy, data were collected between September 2009 and February 2011 in eight medium/small-size organizations, comprising 13 different workplaces as part of a study exploring risk factors of negative acts at work. In each organization, participation was voluntary and confidential. Respondents were instructed to post the questionnaires in a blank envelope in a box at the organization’s Human Resources department or the Occupational Health service. It was agreed with the organizations that no demographic data (except gender) would be collected in order to encourage participation and honesty. As such, 1,500 questionnaires were distributed and 1,151 were returned (response rate 77%). Most participants were men (52.2% vs. 47.8% women).

In Spain, the procedure was the same as in Italy. Data were collected between September 2009 and November 2010 in three large/medium-size organizations, comprising 15 different workplaces located in the Andalusia region in Spain. Response rates in all participating organizations yielded 70% and higher. Thus, 705 questionnaires could be used for this study. The majority of the respondents were men (63.1% vs. 36.9% women).

### Measures

Exposure to Workplace Bullying was measured in Spain using the reduced version of the Negative Acts Questionnaire-Revised (NAQ-R: Einarsen et al., 2009) developed by Moreno Jiménez et al. (2007) and in Italy we used the validated version (Balducci et al., 2010; Giorgi et al., 2011). Participants scored the frequency [response categories were (1) Never, (2) Now and then, (3) Monthly, (4) Weekly, and (5) Daily] that they had been exposed to 14 specific bullying behaviors within the last 6 months (e.g., “being withheld information which affects your performance”). The internal consistency of the questionnaire in both the Italian and the Spanish samples was satisfactory (α = 0.83 and 0.88, respectively).

Psychological well-being was measured by the 12-item version of the General Health Questionnaire (GHQ: Goldberg, 1992). The GHQ is a self-administered screening instrument for psychiatric disorder in non-clinical populations that provides a more general measure of psychological well-being. We used a 4-point Likert-type scale (0-1-2-3) and, after recoding some inverted items, we used the total score of the scale in the subsequent analyses. The scale obtained a satisfactory internal consistency in both the Italian and the Spanish samples (α = 0.85 and 0.87, respectively). Higher scores of the scale mean less psychological well-being.

Job Satisfaction was assessed by using five items from Hartline and Ferrell (1996) that analyze satisfaction with different dimensions of work (salary/wage, job security, social support, supervision, and global satisfaction) on a scale from 1 (“very dissatisfied”) to 5 (“very satisfied”). Cronbach’s alpha was 0.74 and 0.65 for the Italian and the Spanish samples, respectively.

### Statistical Analysis

Our analysis consisted of three steps. First, descriptive statistics and frequencies were calculated. Second, correlational analyses were conducted to obtain a preliminary idea about the relationships among the variables. Third, we theorized that the relationship between exposure to bullying behaviors and psychological well-being is mediated by the job satisfaction, and this simple mediation model is moderated by the country (moderated mediation).

In order to investigate the moderated mediation proposed, we used the PROCESS macro for SPSS (Hayes, 2012), which allows simultaneous testing of complete models that integrate mediation and moderation to examine the conditional nature of indirect effects, as scholars recommend (e.g., Edwards and Lambert, 2007; Preacher et al., 2007). Additionally, consistent with Aiken and West (1991), we explored the shape of the interaction effect of job satisfaction and country on mental health problems by conducting a simple slope test and by producing a graphical representation of the relationship between job satisfaction and mental health problems for the Italian and the Spanish employees.

## Results

First, we provide some descriptive analysis to give a picture about how workplace bullying is perceived among the two different samples of employees.

Among Italian employees, the prevalence rate of workplace bullying, based on the Bergen estimation method, was 14.9%, considering that a person is a victim of bullying if s/he responded to at least two negative acts with a frequency of 4 or 5 (Mikkelsen and Einarsen, 2001). Moreover, the most frequently experienced negative acts were calculated after considering the percentage of participants that scored 4 or 5 in each item (see also **Table 1**). The most frequent acts included: “someone withholding information which affects your performance” (12%); “having your opinions and views ignored” (10.7%); and “excessive monitoring of your work” (8.1%). The least frequent acts included: “threats of violence or physical abuse or actual abuse” (0.6); “intimidating behavior such as finger pointing, invasion of personal space, etc.,” (0.9); and “having insulting or offensive remarks made about your person” (2.1%).

**Table 1:** Percentage of participants that indicated they had been a target of each negative act on a weekly or daily basis and differences between countries.

Bullying behavior	Italy		Spain		Difference
NAQ-R item	%	Mean (*SD*)	%	Mean (*SD*)	*F*
(1) Withholding information	12.0	2.08 (1.12)	13.3	1.86 (1.17)	16.79^∗∗^
(2) Being humiliated or ridiculed	2.2	1.29 (0.66)	2.2	1.32 (0.66)	ns
(3) Doing work below competence level	7.6	2.03 (1.19)	13.9	1.74 (0.99)	27.43^∗∗^
(4) Spreading of gossip and rumors	6.3	1.64 (0.92)	5.5	1.64 (0.93)	ns
(5) Being ignored, excluded.	5.3	1.30 (0.66)	2.2	1.45 (0.91)	18.10^∗∗^
(6) Having offensive remarks	2.1	1.26 (0.65)	2.6	1.23 (0.60)	ns
(7) Being shouted at	1.4	1.35 (0.71)	2.8	1.30 (0.62)	ns
(8) Intimidating behavior	0.9	1.15 (0.53)	1.4	1.09 (0.43)	4.85^∗^
(9) Repeated reminders of errors	2.4	1.60 (0.81)	4.0	1.43 (0.71)	20.90^∗∗^
(10) Having opinions and views ignored	10.7	1.25 (0.56)	1.4	1.92 (1.09)	306.24^∗∗^
(11) Excessive monitoring of your work	8.1	1.69 (1.03)	7.8	1.54 (1.04)	8.58^∗∗^
(12) Pressure not to claim something	2.5	1.26 (0.65)	2.1	1.29 (0.69)	ns
(13) Being exposed to workload	5.4	1.68 (0.91)	6.3	1.54 (0.87)	10.55^∗∗^
(14) Violence or physical abuse	0.6	1.03 (0.27)	0.4	1.06 (0.35)	ns

*^∗∗^*p* < 0.01, ^∗^*p* < 0.05.*

As respects to the Spanish sample, the prevalence rate of workplace bullying was 15% (Bergen criteria). The most frequently experienced negative acts reported were: “being ordered to do work below your level of competence” (13.9%); “someone withholding information which affects your performance” (13.3%); and “excessive monitoring of your work” (7.8%), whereas the least frequent acts reported were: “threats of violence or physical abuse or actual abuse” (0.4); “intimidating behavior such as finger pointing, invasion of personal space, etc.,” (1.4); and “having your opinions and views ignored” (1.4%).

Taking into account that the prevalence of workplace bullying was similar in both samples, considering the Bergen criteria, it seems reasonable to conclude that hypothesis 1 was supported by data.

In addition, it’s worthwhile to note that the prevalence obtained in this study is much higher than those of studies conducted in Northern European countries (for a review, see Nielsen et al., 2011).

Second, differences between the two samples of employees in workplace bullying, job satisfaction and psychological well-being were examined using t tests (**Table 2**). The Pearson correlation coefficients for zero-order relationships among continuous variables are displayed in **Table 3**. Correlations among the considered constructs support hypotheses 2 and 3.

**Table 2:** Differences among countries in the main variables of the study.

Variables	Country	*N*	Mean	*SD*	*t*
Workplace bullying	Italy	1155	1.47	0.45	0.21
	Spain	688	1.46	0.52	
Job satisfaction	Italy	1155	3.48	0.71	13.54^∗∗^
	Spain	688	3.35	0.69	
Psychological well-being^1^	Italy	1155	10.65	6.08	183.01^∗∗^
	Spain	688	14.43	6.45	

*^1^A higher score means having lower psychological well-being; ^∗∗^*p* < 0.01.*

**Table 3:** Means, standards deviations, and correlations between the variables of the study.

Variables	Mean	*SD*	1	2	3
(1) Workplace bullying	1.47	0.47	-		
(2) Job satisfaction	3.43	0.71	-0.52^∗∗^	-	
(3) Psychological well-being^1^	12.06	6.08	0.48^∗∗^	-0.43^∗∗^	-

*^1^A higher score means having lower psychological well-being; ^∗∗^*p* < 0.01.*

Finally, we theorized that the relationship between exposure to bullying behaviors and psychological well-being is mediated by job satisfaction, and this simple mediation model is moderated by the country (moderated mediation).

In order to investigate the moderated mediation proposed, we used the PROCESS macro for SPSS (Hayes, 2012). This macro allows simultaneous testing of complete models that integrate mediation and moderation to examine the conditional nature of indirect effects, as scholars recommend (e.g., Edwards and Lambert, 2007; Preacher et al., 2007). This approach also implies a bootstrap procedure for estimating indirect effects, which resamples the data multiple times and calculates the statistic of interest. A 95% confidence interval is next created through the bias-corrected percentile method, to test the significance of indirect effects and their difference. Accordingly, indirect effects were assessed using bootstrapping with 5,000 resamplings – as recommended by Hayes (2013) – to generate 95% bias-corrected confidence intervals of both direct and indirect effects.

**FIGURE 1:**
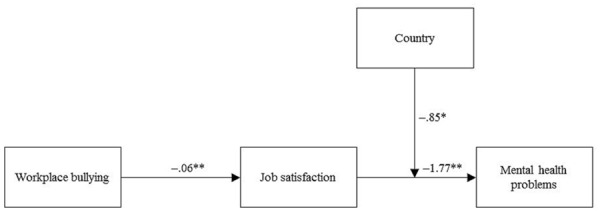
**Standardized path coefficients for the moderated mediation model with job satisfaction as the mediator and country as moderator.** ^∗^*p* < 0.05, ^∗∗^*p* < 0.01.

**Figure 1** shows a path diagram of the results. The results revealed a significant negative relationship between workplace bullying and job satisfaction (β = -0.06, *p* < 0.01) and a negative relationship between job satisfaction and mental health problems (β = -1.77, *P* < 0.01). Additionally, the test of indirect effects revealed that the cross-sectional effect of workplace bullying on mental health problems was mediated by job satisfaction (indirect effect = 0.09, *CI* = 0.08,0.12), which is consistent with our predictions. Next, results showed that the interaction term of job satisfaction and country was significantly associated with mental health problems (β = -0.85, *p* < 0.05). Consistent with Aiken and West (1991), we, therefore, explored the shape of this interaction by conducting a simple slope test and by producing a graphical representation of the relationship between job satisfaction and mental health problems for the two countries.

**FIGURE 2:**
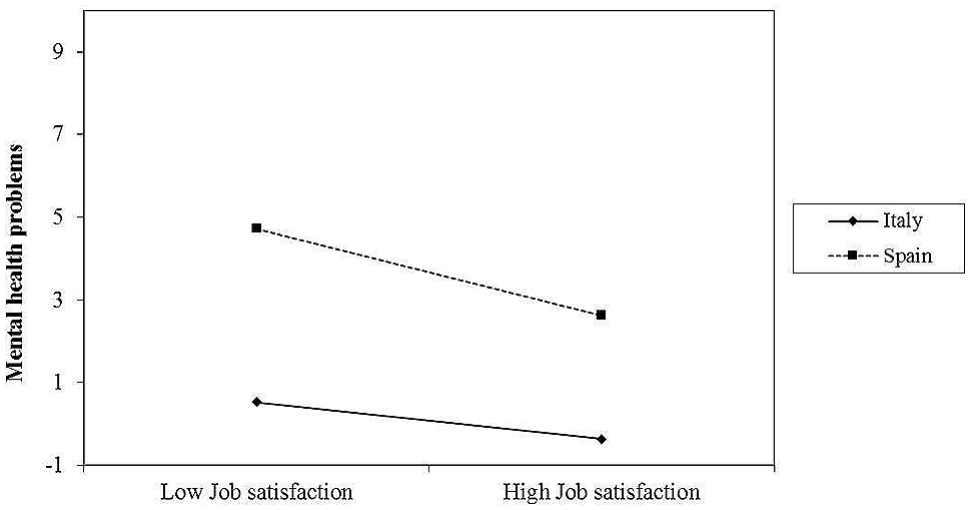
**Interaction between Job satisfaction and country in predicting psychological well-being**.

As can be seen in **Figure 2**, the relationship between job satisfaction and mental health problems was negative and non-significant among the Italian employees (β = -0.64, ns), but it was negative and significant among the Spanish employees (β = -1.5, *p* < 0.01). These results lend preliminary support to hypothesis 4. Likewise, the test of moderated mediation indicated that the indirect effect of workplace bullying on mental health problems via job satisfaction was more positive among the Spanish employees (β = 0.13, *CI* = 0.06,0.11) than among the Italian employees (β = 0.08, *CI* = 0.09,0.16), but this effect was significant for both groups. Additionally, the index of moderated mediation was significant (index = 0.05, *CI* = 0.01,0.09), suggesting that the indirect path from workplace bullying to mental health problems through job satisfaction differed significantly across the two sub-samples. The moderated-mediation hypothesis was, therefore, fully supported.

## Discussion

The present study examines the prevalence rate of workplace bullying among two samples of Italian and Spanish employees and explores the effects of workplace bullying on job satisfaction and psychological well-being.

As such, findings revealed that the prevalence rate of workplace bullying presents similarities among the two samples of employees by using an objective estimation method (operative criterion). Moreover, the prevalence in these samples was much higher than previous findings obtained in Northern European countries by using a similar estimation method (Nielsen et al., 2011). The results obtained with operative criterion (such as NAQ-R) appear to be in contrast with those of the European Foundation for the Improvement of Working and Living Conditions, so, it may be argued that results from previous European Surveys might highlight more awareness and sensitivity about the problem in Northern compared to Southern European countries. This issue should be further investigated through the setting up of national representative samples; in addition, the differences we found in our two samples – not representative of the Italian and Spanish working population – may be related to several factors concerning the composition of the groups.

In addition, as the prevalence of bullying seems high in the sample studied, we could argue that Italian and Spanish employees might tolerate and accept negative acts. As suggested by Giorgi (2012), organizations might implement collective coping responses (Lansisalmi et al., 2000) to negative acts, especially when there is high prevalence of bullying, as in this study.

However, a better understanding of the cultural factors that influence the acceptance of bullying behaviors in the workplace appears fundamental as it could help Italian and Spanish organizations to develop policies and training programs to reduce bullying. Presumably, different organizational interventions are needed in cultures whose values render bullying more acceptable and invisible than in countries where cultural values render bullying socially sanctioned or laws/regulations exist. Further research is needed on this issue.

In addition, our results suggest that workplace bullying negatively affects job satisfaction and psychological well-being, which is in line with previous research (Djurkovic et al., 2006; Rodríguez-Muñoz et al., 2009; Einarsen et al., 2011; Lovell and Lee, 2011). However, an interesting finding is that job satisfaction mediates the relationship between workplace bullying and psychological well-being, and this indirect effect depends on the country. Particularly, in our bullying model among Italian employees, the relationship between job satisfaction and psychological well-being was not significant. Rather, this relationship was contingent upon the moderating effect of country. Indeed, results showed that job satisfaction was associated with poorer psychological well-being in both countries, but this relationship was significant only for the Spanish sub-sample. **Figure 2** specifically shows that the Spanish employees who were low in job satisfaction reported significantly higher mental health problems than their Italian counterparts. The test of the moderated mediation hypothesis further showed that the positive indirect effect of workplace bullying on mental health problems was more pronounced among the Spanish employees than among the Italian employees. Taken together, these findings suggest that Italian employees are more likely to be buffered against the impairing effects of workplace bullying and job dissatisfaction on well-being than Spanish employees. However, this issue should be investigated further.

These results follow previous Italian studies in which a curvilinear relationship between bullying and job satisfaction (Giorgi et al., 2015) and well-being (Fadda et al., 2015), respectively, was found. Job satisfaction and mental health might be not so badly affected because, as described earlier, employees might tolerate and accept bullying at work, particularly in the case of Italian employees. In addition, we found differences in job satisfaction perceptions between the samples studied: Italian employees reported to feel more satisfied than Spanish employees. As Sousa-Poza and Sousa-Poza (2000) noted, there are some determinants of job satisfaction that apply to all countries (such as having an interesting job and good relations with management) and others that are country specific (such as pay and job security), independently of those countries sharing cultural values or belonging to the same family of languages (such as Spain, Italy, Portugal, and France). Therefore, being more satisfied with your job, especially with the employment security or stability that your job offers, may buffer the detrimental effects of workplace bullying on psychological well-being.

### Limitations and Future Directions

Taking these results together, it seems that contextual factors may contribute to a better understanding of workplace bullying, its prevalence and its consequences. However, with cross-sectional data, caution must be applied, as causality cannot be inferred. Indeed, in our study we could not collect data longitudinally. Companies in Italy and Spain, as noted earlier, are still reluctant to diagnose and recognize the phenomenon of bullying at work and they did not give us the permission to collect follow-up data. In future research a full longitudinal design is recommended with at least three measurement points. Above controlling changes of values of satisfaction, health and of mediators/moderators, it would be interesting to observe the potential bullying escalation which might have interesting implications for studying deeply its acceptability/tolerance among employees. Moreover, although in our research we used a contextualized perspective, in which we focus on the context in which the relationships among the constructs studied occur, the lack of identifying the time order of effects is critical. Indeed, our results point out that workplace bullying affects negatively job satisfaction and psychological well-being, which is in line with previous research.

However, lower well-being and job dissatisfaction could have led employees to perceive bullying. It is difficult to discount such a possibility when only cross-sectional data are available. Also, the mediation and moderation effects explored in this study might be biased by the lack of a longitudinal design, thereby limiting the accuracy of our results. This is particularly relevant in the case of mediation effects, since methodologists have shown that cross-sectional mediation models are biased relative to the expected causal processes, and that the bias can occur in either direction, depending on the structure of the supposed causal model (Maxwell and Cole, 2007; Maxwell et al., 2011). Consequently, the cross-sectional indirect effect of workplace bullying on wellbeing via job satisfaction that was found in the present study (indirect effect = 0.09, *CI* = 0.08,0.12) might be less likely to appear in longitudinal designs.

Future research should therefore adopt longitudinal design in order to obtain more reliable estimates of the causal effects of workplace bullying. Specifically, fully longitudinal designs, where time elapses both between the measurement of the independent variable and the mediator and between the mediator and the dependent variable, are preferable over half-longitudinal designs, in which two of the three variables are measured concurrently. This is because fully longitudinal designs, unlike half-longitudinal designs, allow testing the stability of indirect effects (i.e., the degree to which individual differences in a variable remain stable over time), which is an essential criteria to draw rigorous inferences about the interpretability and generalizability of indirect effects (Cole and Maxwell, 2003; MacKinnon, 2008; Preacher, 2015).

Nonetheless, consistent with Cerin’s (2010) recommendations, we mitigated the limitations associated with cross-sectional mediation analysis by relying on a large sample, which helps reduce bias in regression estimates due to measurement error (Kline, 2011). Additionally, it is worth mentioning that our findings are consistent with those from prior longitudinal studies. For example, Rodríguez-Muñoz et al. (2009), who estimated cross-lagged effects of workplace bullying on job satisfaction and engagement, showed that there was no significant cross-lagged effect of job-related well-being on bullying at work, suggesting that bullying can be considered as a cause, rather than a consequence of job-related well-being.

Another limitation is that, our samples are not representative of the Italian and Spanish working population, as it would be necessary to investigate prevalence rates of negative behaviors in a well-defined population. Consequently, our results are related exclusively to the two samples we analyzed and the differences observed may have been influenced by the characteristics of the composition of the groups. Finally, our study used only one source of information for data gathering (self-reported questionnaire), which might introduce common method bias and inflate correlations between variables (Podsakoff et al., 2003). Thus, further research should overcome these limitations and conduct studies with different information sources, such as objective outcome variables (e.g., sickness absence data, other physical or mental disorders, and physiological measures; Hansen et al., 2006), and representative samples.

Further studies may also consider directly assessing cultural variables, since the Negative Acts Questionnaire may be “culturally biased” in the sense that some negative acts may be more frequent and/or perceived as more severe in some cultures than in others (Hoel et al., 2004; Escartín et al., 2009, 2011). Research questions that could be asked include whether: feminine-oriented cultures or with less power distance will report less occurrence of bullying; some negative acts, such as “intimidating behavior,” will be more frequent in masculine than feminine societies; or cultures with a higher score in power distance will report more situations of vertical bullying than horizontal bullying. In addition, the impact of cultural dimensions, such as assertiveness and in-group collectivism, on organizational bullying should be considered, as advocated by the literature (Jacobson et al., 2014).

### Practical Implications and Conclusion

A contextualized approach in the study of workplace bullying, which appears so widespread in the sample studied, may have important implications for attaining a better comprehension about the phenomenon, but also for conducting interventions to deal with workplace bullying.

On the one hand, cross-cultural comparisons among countries seem needed. The use of validated questionnaire is suggested for these purposes. In this sense, the use of objective methods, such as the Negative Acts Questionnaire and the operational criterion, would be useful to establish bullying prevalence and to make comparisons among countries. In addition, a focus on bullying among employees in the Southern European countries is very important, since the quality of working life is currently lower than before and this situation might stimulate higher bullying acceptability and tolerance (Giorgi et al., 2015).

On the other hand, our results show an indirect link between workplace bullying and psychological well-being through job satisfaction, and how this relationship is moderated by the country. If we consider that job satisfaction could have a mediating effect on how workplace bullying affects psychological well-being, then we should intervene in those aspects determining job satisfaction (especially those which are specific for a certain country). Additionally, a more macro-level analysis, such as the one followed in this paper, should be taken into account when specific interventions are developed to counteract workplace bullying. It can be argued that anti-bullying policies need to be sensitive to the cultural context in which they are applied, since the nature of bullying behaviors may differ among countries and there could be a different tolerance of bullying behaviors across cultures.

## Conclusion

The current paper explores in more depth the negative consequences of workplace bullying on job satisfaction and well-being, taking a contextualized approach into account, comparing the perceptions of employees in two samples of Southern European countries with traditionally less awareness of this phenomenon. Our results show that, even though there are no differences in bullying prevalence in the samples studied, the negative consequences vary depending on the country, indicating that work outputs (such as salaries, benefits, status, working conditions, intrinsic aspects) are important determinants to explain the impact of workplace bullying.

## Conflict of Interest Statement

The authors declare that the research was conducted in the absence of any commercial or financial relationships that could be construed as a potential conflict of interest.
